# Supplementary material for: Role and mechanism of NCAPD3 in promoting malignant behaviors in gastric cancer
Source: Front Pharmacol. 2024 Apr 22;15:1341039. doi: 10.3389/fphar.2024.1341039 (PMC11070777; doi:10.3389/fphar.2024.1341039)
Supplement: Supplementary file 11 [file DataSheet2.ZIP › GSEA/Transcipt factor/my_analysis.Gsea.1599461814734/ELF2_TARGET_GENES.html]

Details for gene set ELF2\_TARGET\_GENES[GSEA]

|  || Dataset | filtered\_dataset.sample\_info.cls#WT\_versus\_NCAPD3\_MUT |
| Phenotype | sample\_info.cls#WT\_versus\_NCAPD3\_MUT |
| Upregulated in class | WT |
| GeneSet | ELF2\_TARGET\_GENES |
| Enrichment Score (ES) | 0.28853217 |
| Normalized Enrichment Score (NES) | 1.8236755 |
| Nominal p-value | 0.0015822785 |
| FDR q-value | 0.3143659 |
| FWER p-Value | 0.969 |
Table: GSEA Results Summary

  

Fig 1: Enrichment plot: ELF2\_TARGET\_GENES      
 Profile of the Running ES Score & Positions of GeneSet Members on the Rank Ordered List

  

| SYMBOL | TITLE | RANK IN GENE LIST | RANK METRIC SCORE | RUNNING ES | CORE ENRICHMENT || 1 | 91893 | FDXACB1 | 61 | 0.869 | -0.0248 | Yes |
| 2 | 117145 | THEM4 | 65 | 0.860 | -0.0065 | Yes |
| 3 | 55794 | DDX28 | 68 | 0.856 | 0.0125 | Yes |
| 4 | 27304 | MOCS3 | 76 | 0.844 | 0.0274 | Yes |
| 5 | 153769 | SH3RF2 | 82 | 0.824 | 0.0434 | Yes |
| 6 | 55343 | SLC35C1 | 92 | 0.804 | 0.0559 | Yes |
| 7 | 51512 | GTSE1 | 127 | 0.774 | 0.0490 | Yes |
| 8 | 22879 | MON1B | 151 | 0.735 | 0.0494 | Yes |
| 9 | 83877 | TM2D2 | 173 | 0.704 | 0.0505 | Yes |
| 10 | 5189 | PEX1 | 200 | 0.676 | 0.0473 | Yes |
| 11 | 79230 | ZNF557 | 201 | 0.675 | 0.0634 | Yes |
| 12 | 7024 | TFCP2 | 204 | 0.671 | 0.0780 | Yes |
| 13 | 348801 | LNP1 | 214 | 0.664 | 0.0871 | Yes |
| 14 | 22948 | CCT5 | 215 | 0.663 | 0.1030 | Yes |
| 15 | 55599 | RNPC3 | 227 | 0.652 | 0.1104 | Yes |
| 16 | 23247 | KIAA0556 | 241 | 0.638 | 0.1159 | Yes |
| 17 | 161582 | DYX1C1 | 253 | 0.626 | 0.1227 | Yes |
| 18 | 10464 | PIBF1 | 264 | 0.622 | 0.1301 | Yes |
| 19 | 25973 | PARS2 | 266 | 0.620 | 0.1442 | Yes |
| 20 | 403313 | PLPP6 | 274 | 0.615 | 0.1537 | Yes |
| 21 | 80124 | VCPIP1 | 282 | 0.607 | 0.1630 | Yes |
| 22 | 54758 | KLHDC4 | 292 | 0.598 | 0.1705 | Yes |
| 23 | 129831 | RBM45 | 310 | 0.587 | 0.1719 | Yes |
| 24 | 100529261 | CHURC1-FNTB | 343 | 0.570 | 0.1616 | Yes |
| 25 | 23660 | ZKSCAN5 | 345 | 0.570 | 0.1744 | Yes |
| 26 | 9617 | MTRF1 | 349 | 0.568 | 0.1858 | Yes |
| 27 | 2965 | GTF2H1 | 356 | 0.563 | 0.1948 | Yes |
| 28 | 51535 | PPHLN1 | 361 | 0.560 | 0.2052 | Yes |
| 29 | 54093 | SETD4 | 377 | 0.548 | 0.2071 | Yes |
| 30 | 10618 | TGOLN2 | 383 | 0.547 | 0.2164 | Yes |
| 31 | 10212 | DDX39A | 389 | 0.543 | 0.2256 | Yes |
| 32 | 54554 | WDR5B | 395 | 0.539 | 0.2348 | Yes |
| 33 | 83932 | SPRTN | 404 | 0.535 | 0.2416 | Yes |
| 34 | 25917 | THUMPD3 | 412 | 0.530 | 0.2491 | Yes |
| 35 | 84274 | COQ5 | 419 | 0.523 | 0.2571 | Yes |
| 36 | 8607 | RUVBL1 | 451 | 0.506 | 0.2460 | Yes |
| 37 | 25880 | TMEM186 | 462 | 0.498 | 0.2504 | Yes |
| 38 | 10785 | WDR4 | 465 | 0.495 | 0.2608 | Yes |
| 39 | 53944 | CSNK1G1 | 485 | 0.484 | 0.2581 | Yes |
| 40 | 84261 | FBXW9 | 491 | 0.477 | 0.2658 | Yes |
| 41 | 4191 | MDH2 | 494 | 0.476 | 0.2757 | Yes |
| 42 | 6742 | SSBP1 | 501 | 0.472 | 0.2825 | Yes |
| 43 | 54920 | DUS2 | 509 | 0.470 | 0.2885 | Yes |
| 44 | 55245 | UQCC1 | 580 | 0.434 | 0.2466 | No |
| 45 | 23786 | BCL2L13 | 588 | 0.432 | 0.2517 | No |
| 46 | 6208 | RPS14 | 604 | 0.421 | 0.2505 | No |
| 47 | 8021 | NUP214 | 611 | 0.417 | 0.2560 | No |
| 48 | 54663 | WDR74 | 628 | 0.408 | 0.2538 | No |
| 49 | 10280 | SIGMAR1 | 655 | 0.399 | 0.2439 | No |
| 50 | 55157 | DARS2 | 686 | 0.385 | 0.2307 | No |
| 51 | 10452 | TOMM40 | 696 | 0.380 | 0.2330 | No |
| 52 | 54851 | ANKRD49 | 705 | 0.373 | 0.2360 | No |
| 53 | 55122 | AKIRIN2 | 721 | 0.363 | 0.2334 | No |
| 54 | 23640 | HSPBP1 | 735 | 0.359 | 0.2323 | No |
| 55 | 58509 | CACTIN | 748 | 0.351 | 0.2317 | No |
| 56 | 9238 | TBRG4 | 786 | 0.330 | 0.2120 | No |
| 57 | 2224 | FDPS | 841 | 0.273 | 0.1781 | No |
| 58 | 1984 | EIF5A | 846 | 0.259 | 0.1813 | No |
| 59 | 8738 | CRADD | 922 | -0.349 | 0.1336 | No |
| 60 | 25901 | CCDC28A | 946 | -0.366 | 0.1251 | No |
| 61 | 55148 | UBR7 | 987 | -0.396 | 0.1047 | No |
| 62 | 51343 | FZR1 | 1005 | -0.407 | 0.1017 | No |
| 63 | 53349 | ZFYVE1 | 1018 | -0.417 | 0.1027 | No |
| 64 | 11152 | WDR45 | 1032 | -0.430 | 0.1033 | No |
| 65 | 100534599 | ISY1-RAB43 | 1082 | -0.462 | 0.0777 | No |
| 66 | 100533483 | DYX1C1-CCPG1 | 1127 | -0.500 | 0.0568 | No |
| 67 | 11132 | CAPN10 | 1141 | -0.508 | 0.0592 | No |
| 68 | 83548 | COG3 | 1211 | -0.588 | 0.0217 | No |
| 69 | 22931 | RAB18 | 1231 | -0.608 | 0.0220 | No |
| 70 | 2005 | ELK4 | 1235 | -0.612 | 0.0344 | No |
| 71 | 114907 | FBXO32 | 1248 | -0.625 | 0.0404 | No |
| 72 | 91947 | ARRDC4 | 1282 | -0.671 | 0.0318 | No |
| 73 | 26959 | HBP1 | 1395 | -1.073 | -0.0263 | No |
| 74 | 255394 | TCP11L2 | 1408 | -1.538 | 0.0015 | No |
Table: GSEA details [plain text format]

  

Fig 2: ELF2\_TARGET\_GENES      
 Blue-Pink O' Gram in the Space of the Analyzed GeneSet

  

Fig 3: ELF2\_TARGET\_GENES: Random ES distribution      
 Gene set null distribution of ES for **ELF2\_TARGET\_GENES**
